# Enhanced Electromagnetic Absorption Properties of Commercial Ni/MWCNTs Composites by Adjusting Dielectric Properties

**DOI:** 10.3389/fchem.2020.00097

**Published:** 2020-02-28

**Authors:** Pei-Yan Zhao, Hui-Ya Wang, Guang-Sheng Wang

**Affiliations:** School of Chemistry, Beihang University, Beijing, China

**Keywords:** Ni/MWCNTs, dielectric properties, microwave absorption, filling content, impedance matching

## Abstract

In this manuscript, we constructed a Ni/MWCNTs absorber and properly adjusted the permittivity resulted from absorber content in the PVDF to optimize impedance matching properties. Both ε′ and ε″ increase obviously with the increasing content of Ni/MWCNTs in PVDF, demonstrating that dielectric properties are dependent on the conductivity. Moderate dielectric properties and excellent impedance matching can be obtained for the filler content of 20 wt% Ni/MWCNTs. Reasonable impedance matching allows electromagnetic waves to propagate into the materials and finally realize energy dissipation through dielectric loss and interfacial polarization. As expected, the minimum reflection loss (*RL*) of −46.85 dB at 6.56 GHz with a low filler loading (20 wt%) and wide effective bandwidth (*RL*<-10 dB) of 14.0 GHz in the thickness range of 1.5–5.0 mm was obtained for the commercial Ni/MWCNTs composites, which is promising for mass production in industrial applications. Our findings offer an effective and industrialized way to design high-performance material to facilitate the research in microwave absorption.

## Introduction

Nowadays, electromagnetic interference and pollution have become a potential hazard to the normal operation of electronic equipment and human health, accompanied by the massive usage of electronic devices in civil and military applications (Yin et al., 2016; Fang et al., 2017; Yang et al., 2018). It is in urgent need to exploit electromagnetic wave absorbers, which possess excellent absorption ability and wide effective band at a relatively thin thickness (Zhou et al., 2017). Based on the loss mechanism, microwave absorbing materials can be usually divided into dielectric loss that mainly attenuated energy through polarization effects and magnetic loss materials, which dissipated energy by resonances and eddy current effects (Yan et al., 2017; Hu P. P. et al., 2019). Carbon materials, dielectric loss materials, have been proved as effective microwave absorption materials due to their superior electronic properties, large specific surface area, and low density (Zhao et al., 2016; Liu Y. et al., 2018; Wang Y. et al., 2019). In particular, carbon nanotubes (CNTs) with unique one-dimensional tubular nanostructure can enhance interface polarization and offer enormous sites for electromagnetic wave scattering, which contributed to electromagnetic wave attenuation (Chen et al., 2017). However, it is difficult for unilateral loss materials to achieve a desired microwave absorption on account of the poor impedance matching, which greatly limits their applications (Wang L. et al., 2019).

Accordingly, incorporation of dielectric loss materials with magnetic loss media may be an ideal method to further enhance the microwave attenuation properties (Xu J. et al., 2018; Xu et al., 2019). Till now, composites based on CNTs and various magnetic nanoparticles have been extensively utilized for microwave absorbing materials (Wang et al., 2016; Kuang et al., 2019). Hu Q. et al. (2019) constructed NiCo_2_O_4_@CNTs hybrid sponges via a facile hydrothermal method and heat treatment. The resultant composites possess a minimum reflection loss of −45.1 dB and an effective absorption bandwidth of 4.1 GHz. Chen et al. (2015) reported a novel 3D Fe_3_O_4_-MWCNTs nanostructures with enhanced tunable microwave absorption. When the thickness was 6.8 mm, the minimum reflection loss value of −23.0 dB and −52.8 dB were observed at 4.1 GHz and 12.8 GHz, which are superior to those of pure MWCNTs as well as other hybrids of Fe_3_O_4_. As expected, the dielectric–magnetic matching components greatly contribute to the improvement of absorbing properties.

Compared with traditional ferrite absorbing materials, transition metal nickel has a higher saturation magnetization value (55 emu/g at room temperature) and Snoek limit (high natural resonance of 8.23 GHz), allowing a high relative permeability in high-frequency range to achieve good impedance matching (Liu D. et al., 2018; Xie P. et al., 2018). Ning et al. (2018) prepared heterostructural Ni/N-CNTs by a modified one-step pyrolysis process, which demonstrated promising candidates in microwave absorption (MWA) application. The minimum reflection loss of as-synthesized samples with 10 wt% loading is up to −34.1 dB and the effective absorption bandwidth is as wide as 4.72 GHz, which benefits from the optimized impedance matching and the intense dielectric relaxation. Zhang et al. (2019c) successfully synthesized special hierarchical yolk-shell ZnO-Ni@CNT microspheres by controlling pyrolysis of the Zn-Ni bimetallic metal-organic framework, which displayed a minimum reflection loss value of −58.6 dB at 2.3 mm, and the effective absorption frequency range of 4.8 GHz. Such superior performance of ZnO-Ni@CNT microspheres benefited from well-matched impedance, special porous hierarchical structure, interfacial polarization, conductive loss, and multiple reflections. Although these works improved the microwave absorption properties to a certain extent, low yield suppresses the development and practical applications. It remains a great challenge to prepare the absorbers with high efficiency and wide bandwidth in large scales.

The commercial Ni/MWCNTs composites with high yield are conducive to prepare the absorbers with high efficiency and wide bandwidth in large scales. In addition, PVDF has been widely utilized in modern electronic appliances because of its compact size, superior hydrophobicity, anticorrosion resistance, and excellent flexibility. Therefore, we purchased Ni/MWCNTs material and adjusted filler loading in PVDF matrix to obtain enhanced microwave absorption property that can be widely used. As known, a moderate conducting material is suitable as an absorbing material, whereas material with high conductivity can be a promising candidate as an electromagnetic shield. In this context, varying the content of Ni/MWCNTs in PVDF is an efficient method to regulate permittivity and impedance matching, thus imparting it with the potential for desired absorbing property. Moreover, the unique one-dimensional tubular nanostructure offers tremendous sites for electromagnetic wave scattering. A remarkable reflection loss of −46.85 dB and a broad bandwidth of 3.2 GHz are achieved by tailoring the filler content with 20 wt%.

## Experimental

### Materials

The Ni/MWCNTs (mean diameter 50 nm, length <10 μm, and Ni content of 60 wt%) were purchased from Nanjing Xianfeng technology co. Ltd and used without further treatment. The commercial Ni/MWCNTs powder was prepared by electroplating nickel on the surface of CNTs prepared by chemical vapor deposition method. Polyvinylidene fluoride (PVDF) and *N, N*-dimethylformyl (DMF) were supplied by Beijing Chemical Factory (Beijing, China).

### Characterization

The crystalline structure of the Ni/MWCNTs was characterized using X-ray powder diffraction (XRD) on a Rigaku Dmax 2200 diffractometer with Cu Kα radiation (λ = 1.5416 Å). The morphology and microstructures were analyzed by a scanning electron microscope (SEM, Quanta 250 FEG) a field emission scanning electron microscope (FESEM, JEOL JSM-7500F), and a transmission electron microscope (TEM, JEOL JEM-2100F). Raman spectroscopy (Horiba Jobin Yvon, LabRAM HR800) was used to record the properties of samples in the range of 200–2,000 cm^−1^ with an excitation wavelength of 514.5 nm. The chemical composition of the samples was examined by X-ray photoelectron spectroscopy (XPS) using ESCA Lab MKII X-ray photoelectron spectrometer. The magnetic properties were carried out on a Lakeshore Vibrating Sample Magnetometer (VSM, Riken Denshi Co. Ltd, Japan).

### Measurements of Electromagnetic Parameters

The relative complex permeability and permittivity were measured by a vector network analyzer (Agilent, PNA 5244A). Before test, Ni/MWCNTs and PVDF were mixed in DMF with different mass fractions evenly and dried to form a film, which was then being pressed into a ring-like compact structure (with a 3.04-mm inner diameter and a 7.00-mm outer diameter).

## Results and Discussion

**Figure 1:**
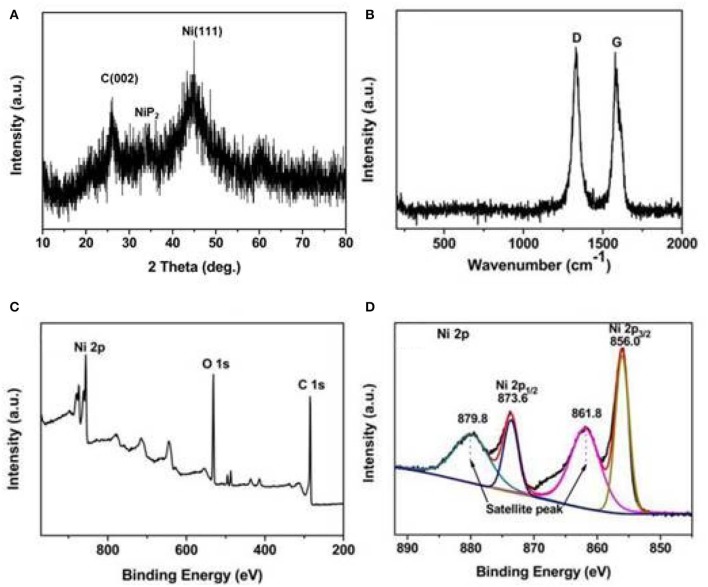
XRD patterns **(A)**, Raman spectra **(B)**, and XPS spectra of survey spectrum **(C)**, and Ni 2p **(D)** of Ni/MWCNTs composites.

The crystal structure of the Ni/MWCNTs composite was measured by XRD, as displayed in Figure 1A. The diffraction peak at 26.1° can be assigned to the (002) reflection of the MWCNTs, indicating that the CNTs structure was not destroyed (Zhao et al., 2010). It is noted that other diffraction peaks located at 2θ = 33.8° and 44.8° can be indexed to the NiP_2_ and metallic Ni (111) structure, respectively (Kim et al., 2014; Yim et al., 2016). The Raman spectroscopy was also tested to aid in the investigation of the compositions. As can be seen in Figure 1B, the existence of MWCNTs is clearly confirmed by the featured D band at 1,330.3 cm^−1^ and D band at 1,581.9 cm^−1^. D peak is related to disordered structure of amorphous carbon, forbidden in complete graphitization and became active in the presence of disordered or finite size crystals of graphite, while the G peak corresponds to the E_2*g*_ mode of the telescopic vibration of the *sp*^2^ bond (Ferrari and Robertson, 2000; Cheng et al., 2018). Meanwhile, the intensity ratio of D band to G band (*I*_*D*_*/I*_*G*_) can be utilized to assess the disorder degree of carbon materials. A high *I*_*D*_*/I*_*G*_ value of the Ni/MWCNTs composite suggested the presence of numerous defects in the graphitized structure or the edges, which may have contributed to induce dipole/electron polarization (Wen et al., 2014). The information and atomic structure were obtained by the XPS technique. The survey scan displayed in Figure 1C confirms the presence of Ni, C, and O elements in the composites. In the Ni *2p* spectrum of the sample (Figure 1D), the two satellite peaks of Ni that are located at 861.8 and 879.8 eV can be observed (Fang et al., 2019). Besides, two peaks with a binding energy of 856.0 and 873.6 eV are assigned to Ni^2+^ *2p*_3/2_ and Ni^2+^ *2p*_1/2_, respectively (Zhang et al., 2019a). The result demonstrates that Ni–C or Ni–O–C bonds exist on the Ni/MWCNTs surface, which is presumably attributed to a partial oxidation of surface metal Ni species (Yang et al., 2019).

**Figure 2:**
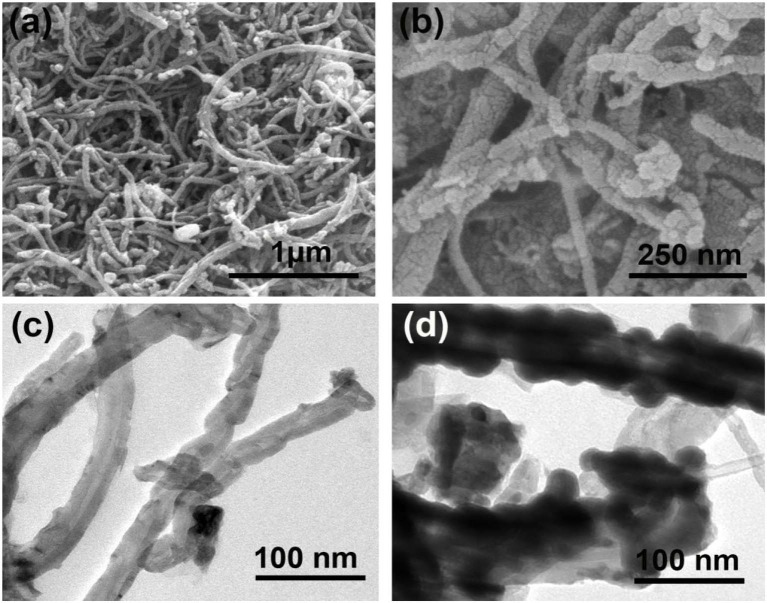
SEM **(A,B)** and TEM **(C,D)** image of Ni/MWCNTs composites.

To further investigate the microstructure and morphology of Ni/MWCNTs composites, SEM and TEM were conducted and shown in Figure 2. As observed from Figures 2A,B, Ni nanoparticles were anchored unevenly to the surface of the MWCNTs, indicating the successful synthesis of Ni/MWCNTs composites. The Ni/MWCNTs composites with a diameter of 25–73 nm and length up to several micrometers are aggregated into a porous three-dimensional network. The TEM image in Figures 2C,D further indicates that conductive MWCNTs can serve as the skeleton to deposit Ni nanoparticles with an obvious particle accumulation, and then self-assemble into irregular nanoparticles.

Magnetic hysteresis loops of Ni/MWCNTs composite were conducted by a vibrating sample magnetometer (VSM) at room temperature to investigate magnetic properties. As seen from Figure S1, typical S-type M-H curve indicates ferromagnetic behavior with a saturating reversible magnetization. The saturation magnetization (*Ms*) of Ni/MWCNTs is 0.76 emu/g, which is lower than bulk nickel (55 emu/g) and elemental nickel reported in other literatures (42 emu/g) (Liu et al., 2016). Such a decline can be attributed to the existence of nonmagnetic carbon with low crystallinity.

**Figure 3:**
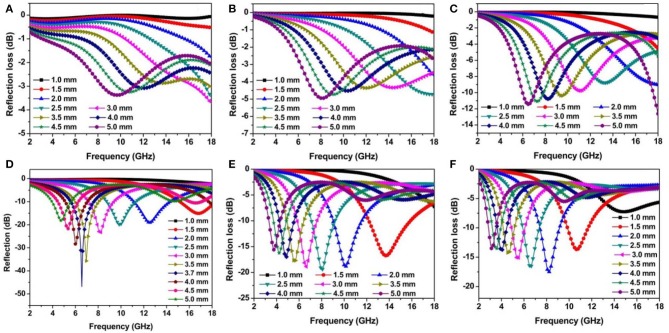
**(A–F)** Frequency dependence of *RL* curves for the PVDF composites with 1, 5, 10, 20, 30, and 40 wt% Ni/MWCNTs at various thicknesses.

The microwave absorption properties, in terms of reflection loss (*RL*), are calculated from the relative permeability and permittivity through the following transmission line theory (Xie et al., 2017; Wang et al., 2018):

(1) $$\begin{array}{l} RL=20\text{ log}\left|\frac{Z_{in}-1}{Z_{in}+1}\right| \end{array}$$

(2) $$\begin{array}{l} Z_{in}=Z_{0}\sqrt{\frac{\mu_{r}}{\varepsilon_{r}}\tanh\left(j\frac{2\pi fd}{c}\sqrt{\mu_{r}\varepsilon_{r}}\right)} \end{array}$$

where *Z*_0_ and *Z*_*in*_ are the intrinsic impedance of free space and the input impedance of the absorber, respectively. *d, c*, and *f* represent the thickness of absorber, the velocity of light, and the frequency of electromagnetic wave, respectively. Commonly, the excellent microwave absorption materials are required to have a *RL* value lower than −10 dB within a wide bandwidth under thin thickness. Figure 3 presents the *RL* curves of Ni/MWCNTs absorber with different loading contents in the frequency range of 2–18 GHz. It is clear that the microwave absorption property of Ni/MWCNTs composite is significantly enhanced initially and then decreases with the increase of filler loading. The minimum *RL* values shift to a lower-frequency range with the increases of Ni/MWCNTs filler loadings, which can be reasonably ascribed to interfacial polarization that usually occurs in the low-frequency stage. For the composites containing 1 wt% Ni/MWCNTs (Figure 3A), it exhibits poor microwave absorption capacity with the *RL* value of −1.6 dB over the tested frequency of 2–18 GHz. Such poor absorption property also emerges in other filler loading of 5 wt and 10 wt%, which is mainly originated from the weak dielectric loss derived from low conductivity and interfacial polarization (Figures 3B,C). However, when the filler loading is 20 wt%, the minimum *RL* value of the materials reaches −46.85 dB at 6.56 GHz with a thickness of 3.7 mm, and the effective bandwidth is up to 3.20 GHz (Figure 3D). Besides, the *RL* value exceeding −10 dB can be achieved in the range of 4.0–18.0 GHz by varying thickness from 1.5 to 5.0 mm. As for sample with 30 wt and 40 wt% filler contents shown in Figures 3E,F, it exhibits poor absorbing performance because of the high conductivity brought by the formation of a conductive network, which makes electromagnetic waves reflect on the surface of materials. Thus, the samples can achieve better microwave absorption by adjusting the absorber thickness and filler loading.

Moreover, it should be pointed out that the optimal *RL* peaks are shifted toward lower frequency along with the change of thickness from 1.0 to 5.0 mm. This interesting phenomenon is usually expressed by the 1/4 wavelength cancellation equation (Liu H. et al., 2018):

(3) $$\begin{array}{l} t_{m}=\frac{nc}{4f_{m}\sqrt{\varepsilon_{r}\mu_{r}}} \end{array}$$

Obviously, the peak frequency is inversely proportional to the thickness of absorbers. As shown in Figure S2, this sample obeys the λ/4 model, meaning the reflected electromagnetic waves from both the air–absorber interface and the absorber–metal background interface are out of phase by 180°, making them cancel out and then resulting in a minimum *RL* value (Lou et al., 2018).

**Figure 4:**
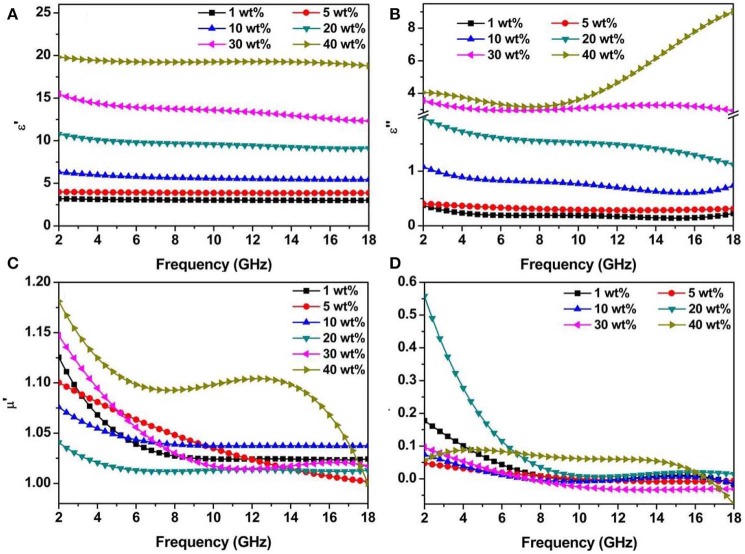
Electromagnetic parameters of samples with different filler loading in the frequency range of 2–18 GHz, real part of permittivity **(A)**, imaginary part of permittivity **(B)**, real part of permeability **(C)**, and imaginary part of permeability **(D)**.

To further investigate the associated wave absorption mechanisms and influence of the filler content on microwave absorption properties, frequency-dependent complex permittivity and permeability were examined in 2–18 GHz for Ni/MWCNTs sample loading from 1 wt to 40 wt%. As known, the complex relative permittivity (ε_*r*_) and permeability (μ_r_) highly determine reflection and attenuation characteristics of absorbers. In general, the real permittivity (ε′) and permeability (μ′) stand for the storage capability of the electric and magnetic energy, while the imaginary parts (ε″ and μ″) correspond to the dissipation capability (Xie A. et al., 2018). As shown in Figures 4A,B, the ε′ values of six different loading samples over 2–18 GHz present a declining trend with a certain degree of fluctuations from 3.20 to 2.99, 3.99 to 3.86, 6.33 to 5.43, 10.77 to 9.07, 15.49 to 12.32, and 19.81 to 18.78, respectively. Both ε′ and ε″ increase obviously with the increasing content of Ni/MWCNTs in PVDF, demonstrating that dielectric properties are dependent on the conductivity (Duan et al., 2018). The increase is mainly determined by the interfacial polarization and enhanced conductivity resulted from the gradually establishment of a large conductive networks as the increasing filler loading (Figure S5), which is beneficial for electron transport and space charge polarization (Xu W. et al., 2018; Wu et al., 2019). Based on the free electron theory (Liang et al., 2016; Guan et al., 2018; Lu et al., 2019): $\sigma =2\pi f\varepsilon^{\prime \prime }\varepsilon_{0}$, the frequency-dependent conductivity profiles were characterized in Figure S3. The conductivity values of 0.04–0.73 S/m are obtained for the Ni/MWCNTs composite with the loading of 1, 5, and 10 wt% in 2–18 GHz. For the filler content of 40 wt%, it shows much larger values from 0.45 to 0.92 S/m in the same frequency range. Undoubtedly, the ameliorated conductivity will enhance electromagnetic performance, but the high electrical conductivity leads to the imbalance of impedance matching and weakens microwave absorption property. As observed in Figures 4C,D, the μ′ and μ″ values of different filler samples exhibit pretty similar variation trends, showing a sharp decrease and then keep relatively flat with a slight fluctuation in 8.0–18.0 GHz. This is mainly ascribed to the stronger eddy current loss in alternating the EM field at high frequency. Furthermore, derivative internal magnetic field can be induced by an alternating current electric field generated by the eddy current (Qiu et al., 2016). At high frequency, the loss capability of Ni nanoparticles may be gradually counteracted with the consolidation of the internal magnetic field, resulting in the decreased complex permeability (Liu et al., 2019).

Meanwhile, the dielectric and magnetic loss tangents of Ni/MWCNT composites with different filler loading are calculated to evaluate the attenuation loss and shown in Figure S4. Except for the loading of 40 wt%, the tanδ_ε_ values enhance with the increase of filler proportions (Figure S4A). From Figure 4B, it is clear that tanδ_μ_ is much lower than that of the dielectric loss, which indicates that the dielectric loss has a major contribution to electromagnetic loss. Besides, negative tanδ_μ_ values appear in the high frequency, which indicates that the magnetic energy is radiated out from the Ni/MWCNTs absorbers and converted into electrical energy to increase tanδ_ε_.

Actually, the *RL* values are not simply determined by their dielectric/magnetic loss capability, but more dependent on impedance behavior that is a necessary prerequisite for obtaining excellent microwave absorption performance, as well as overall attenuation ability, which is another key factor to impact the intensity and bandwidth of *RL* peak. Generally, impedance matching and attenuation constant (α) can be expressed by the relative input *Z* = |*Z*_*in*_/*Z*_0_| and deduced using the following equation (Ma et al., 2016; Wu et al., 2018), respectively.

(4) $$\begin{array}{l} \alpha =\frac{\sqrt{2}\pi f}{c}\sqrt{(\mu \prime \prime \varepsilon \prime \prime -\mu \prime \varepsilon \prime )+\sqrt{{\left(\mu \prime \prime \varepsilon \prime \prime -\mu \prime \varepsilon \prime \right)}^{2}+{\left(\mu \prime \varepsilon \prime \prime +\mu \prime \prime \varepsilon \prime \right)}^{2}}} \end{array}$$

**Figure 5:**
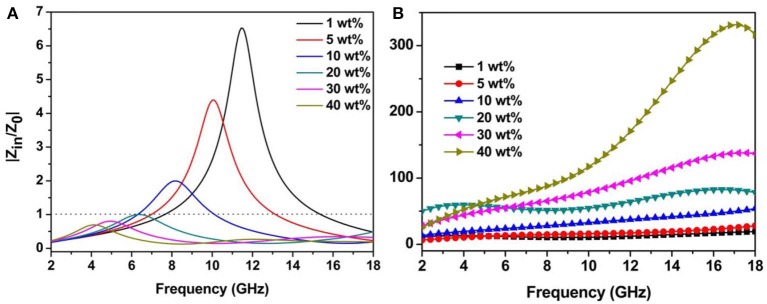
Modulus of normalized input impedance Z **(A)** of 3.7 mm and attenuation constant α **(B)** of Ni/MWCNT composites with different filler loading.

It can be found from Figure 5 that the *Z* values decrease with the increasing filler contents, whereas the attenuation constant of samples increase as the filler contents increase in PVDF. As for the sample with 20 wt% loading, the |*Z*_*in*_/*Z*_0_| values are the nearest to 1 and even coincide with dot lines in a certain frequency band (Figure 5A). These results illustrate that the excellent microwave absorption property has also been caused by the superior impedance matching in the sample with 20 wt% loading. The sample with 40 wt% loading possesses best attenuation property but poor impedance matching behavior, thus leading to that only very limited incident electromagnetic waves can be transmitted into absorber. In this case, no matter how good the attenuation ability they own, it will not create desirable absorption properties.

Based on these results, it can be concluded that the enhanced microwave absorption mechanism may be the well-matched impedance, conductive loss, multiple reflections and scatterings, and synergistic effect of dielectric loss and magnetic loss. The optimal impedance matching means that most of the incident electromagnetic wave can effectively propagate into the absorbers and further be attenuated by the multiple loss mechanism (Liu P. et al., 2017). Ni/MWCNTs composites with unique one-dimensional tubular nanostructure and appropriate conductivity may generate microcurrent and be beneficial to the enhancement of conduction loss (Zhao et al., 2018). Space charge polarization and interfacial polarization induced by space charge accumulation between various interfaces make a contribution to the dielectric loss (Liang et al., 2019). Furthermore, dipole polarization induced by abundant defects also enhance microwave attenuation capacity (Zhang et al., 2019b). Table 1 summarizes the MWA performance of various carbon-based magnetic composites reported in literature. Ni/MWCNTs prepared in this work have the advantages of low filler loading, thinner thickness, and broadening efficient absorption bandwidth (EAB).

**Table 1:** Microwave absorption performances of various carbon-based magnetic composites in previous reports compared with this work.

**Absorber**	**Matrix**	**Content** **(wt%)**	**RL_***min***_** **(dB)**	***d*** **(mm)**	**EAB (GHz)** **(RL < −10 dB)**	**References**
CNTs/NiCo_2_O_4_	Paraffin	30	−45.1	2.5	2.7	Hu Q. et al., 2019
Fe@NCNTs	Paraffin	10	−30.4	3.2	5.7	Ning et al., 2018
Ni@NCNTs	Paraffin	10	−34.1	3.2	4.7	
MWCNT/NiFe_2_O_4_	Paraffin	50	−42.3	1.2	3.8	Zhang et al., 2019b
Porous Ni/C composites	Paraffin	40	−51.8	2.6	3.5	Liu W. et al., 2017
Ni/CNT composites	Paraffin	20	−30.0	2.0	6.5	Sha et al., 2017
Ni/MWCNT	Paraffin	30	−37.9	4.0	3.6	Tong et al., 2014
MoO_3_/MoS_2_ Hybrid	PVDF	20	−38.5	2.0	2.0	Li et al., 2019
C-Fe_3_O_4_/PVDF	PVDF	40	−41.75	3.0	2.01	Adebayo et al., 2019
Ni/MWCNTs	PVDF	20	−46.85	3.7	3.2	This work

## Conclusions

In summary, we created a prominently excellent microwave absorber with moderate conductivity and optimal impedance matching by adjusting the filling content based on purchased Ni/MWCNTs materials. As a result, not only a strong reflection loss (−46.85 dB) but also a broad bandwidth (3.2 GHz) in frequency of 2–18GHz was achieved in the Ni/MWCNTs composites with only 20 wt% fillers. Its excellent absorbing properties are mainly ascribed to well impedance matching and synergistic effect of dielectric loss and magnetic loss. The aforementioned results provide a novel strategy to obtain a commercially available absorber that can be widely applied in many fields.

## Data Availability Statement

All datasets generated for this study are included in the article/Supplementary Material.

## Author Contributions

P-YZ performed the main experimental operation and drafted the manuscript. H-YW performed the data analyses. G-SW contributed to the conception of the study and financial support.

### Conflict of Interest

The authors declare that the research was conducted in the absence of any commercial or financial relationships that could be construed as a potential conflict of interest.
