# Supplementary material for: Enhanced Electromagnetic Absorption Properties of Commercial Ni/MWCNTs Composites by Adjusting Dielectric Properties
Source: Front Chem. 2020 Feb 28;8:97. doi: 10.3389/fchem.2020.00097 (PMC7058662; doi:10.3389/fchem.2020.00097)
Supplement: Supplementary file 1 [file Data_Sheet_1.pdf]

Supplementary Material

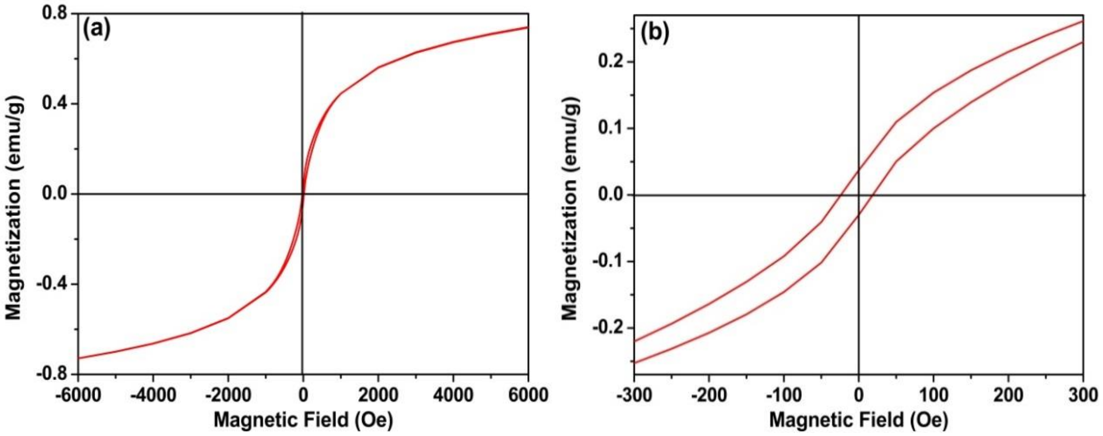

Fig. S1 Magnetic hysteresis loops of Ni/MWCNTs composites (a) and magnification view for coercivity (b).

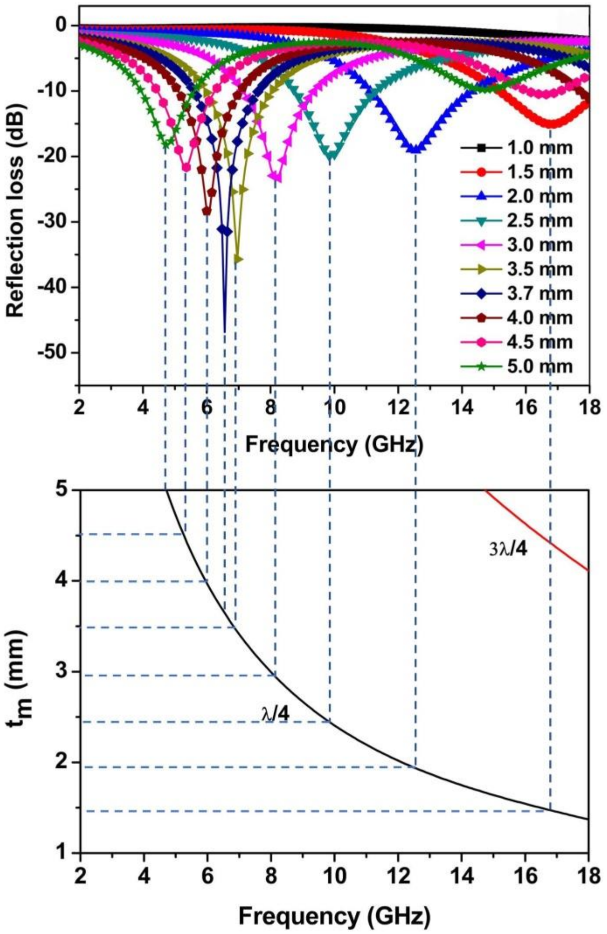

Fig. S2 Dependence of matching thickness (t_m) on matching frequency (f_m) of samples with 20 wt% filler content based on the λ/4, and 3λ/4 models.

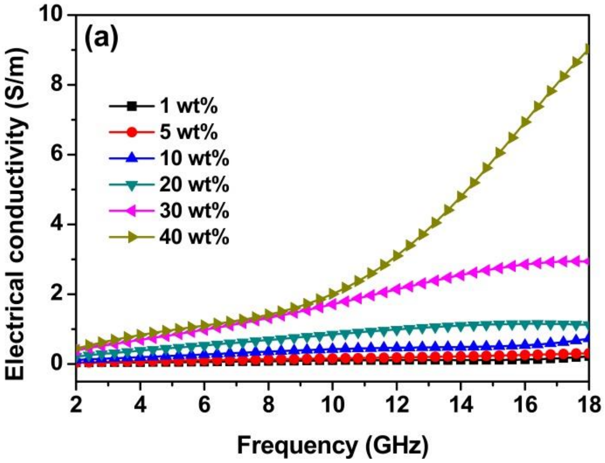

Fig. S3 Frequency dependent electrical conductivity of the Ni/MWCNTs composites with various filler loading.

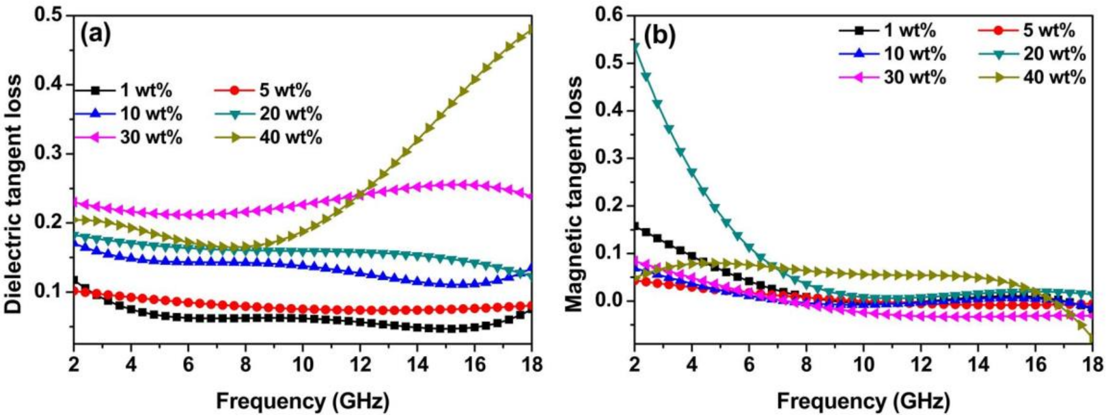

Fig. S4 Frequency dependence on dielectric loss tangents and magnetic loss tangents of samples with different filler loading.

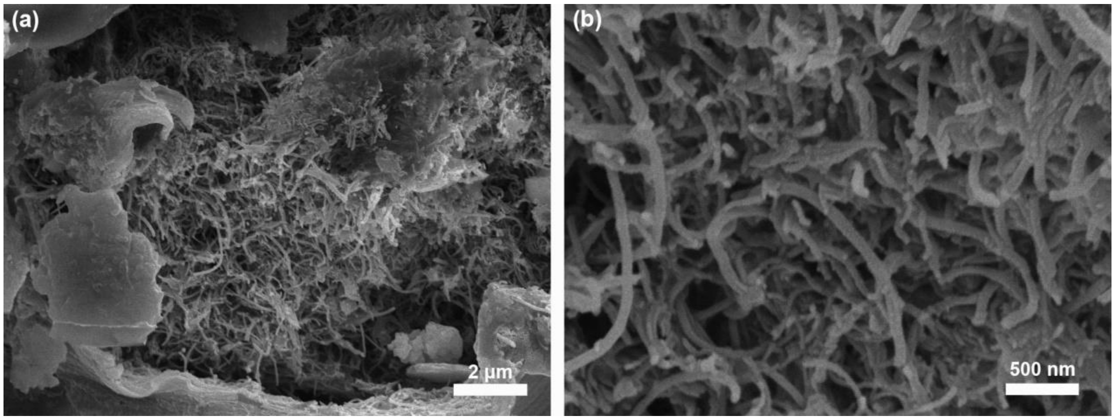

Fig. S5 Cross-sectional SEM images of Ni/MWCNT+PVDF composites with the filler loading of 20 wt%.
